# Exploring Biased Agonism at FPR1 as a Means to Encode Danger Sensing †

**DOI:** 10.3390/cells9041054

**Published:** 2020-04-23

**Authors:** Jieny Gröper, Gabriele M. König, Evi Kostenis, Volker Gerke, Carsten A. Raabe, Ursula Rescher

**Affiliations:** 1 Institute of Medical Biochemistry, Center for Molecular Biology of Inflammation, University of Muenster, Von-Esmarch-Str. 56, D-48149 Muenster, Germany; Jieny.Groeper@ukmuenster.de (J.G.); gerke@uni-muenster.de (V.G.); 2 Cells in Motion” Interfaculty Centre, University of Muenster, 48149 Muenster, Germany; 3 Institute for Pharmaceutical Biology, University of Bonn, Nussallee 6, 53115 Bonn, Germany; G.Koenig@uni-bonn.de (G.M.K.); kostenis@uni-bonn.de (E.K.); 4 Institute of Experimental Pathology, Center for Molecular Biology of Inflammation, University of Muenster, Von-Esmarch-Str. 56, D-48149 Muenster, Germany

**Keywords:** bias analysis, G protein-coupled receptor (GPCR), formyl peptide receptor 1, danger-associated molecular pattern (DAMP), pathogen-associated molecular pattern (PAMP), annexin A1 peptide Ac2-26

## Abstract

Ligand-based selectivity in signal transduction (biased signaling) is an emerging field of G protein-coupled receptor (GPCR) research and might allow the development of drugs with targeted activation profiles. Human formyl peptide receptor 1 (FPR1) is a GPCR that detects potentially hazardous states characterized by the appearance of N-formylated peptides that originate from either bacteria or mitochondria during tissue destruction; however, the receptor also responds to several non-formylated agonists from various sources. We hypothesized that an additional layer of FPR signaling is encoded by biased agonism, thus allowing the discrimination of the source of threat. We resorted to the comparative analysis of FPR1 agonist-evoked responses across three prototypical GPCR signaling pathways, i.e., the inhibition of cAMP formation, receptor internalization, and ERK activation, and analyzed cellular responses elicited by several bacteria- and mitochondria-derived ligands. We also included the anti-inflammatory annexinA1 peptide Ac2-26 and two synthetic ligands, the W-peptide and the small molecule FPRA14. Compared to the endogenous agonists, the bacterial agonists displayed significantly higher potencies and efficacies. Selective pathway activation was not observed, as both groups were similarly biased towards the inhibition of cAMP formation. The general agonist bias in FPR1 signaling suggests a source-independent pathway selectivity for transmission of pro-inflammatory danger signaling.

## 1. Introduction

A key feature of the eukaryotic immune defense is its capability to sense danger signals. Microbes are a source of potentially deleterious infections, and consequently, ‘pathogen-associated molecular patterns’ (PAMPs) and ‘danger-associated molecular patterns’ (DAMPs) represent chemical signatures that are sensed and transduced via the corresponding ‘pattern recognition receptors’ (PRRs) [1,2].

One such molecular pattern is the characteristic N-formylated methionine at the N-terminus of bacterial proteins. This modified amino acid is not utilized for the initiation of eukaryotic protein translation. However, mitochondria, which are considered to represent endosymbionts of bacterial origin [3], initiate protein biosynthesis with N-formylated methionine and might release detectable levels of formylated peptides in situations of enhanced cell death or trauma [4,5]. Therefore, the appearance of N-formylated peptides signals potentially hazardous states caused by either bacterial threats (PAMPs) or tissue destruction (DAMPs).

In higher eukaryotes, this unique pattern is sensed by the family of formyl peptide receptors (FPRs), which belong to the superfamily of G protein-coupled receptors (GPCRs) [5,6]. Thus, a shared sensor system detects bacteria-derived ligands and those that are liberated by non-infectious tissue destruction from mitochondria. The corresponding formylated peptides elicit strong signals via the activation of formyl peptide receptor 1 (FPR1) [7,8]. Here, we addressed whether human FPR1 is capable to decode the actual source of threat via different, i.e., ligand-specific signaling, and consequently modify the elicited cellular responses. We hypothesized that these layers of signal information are encoded by distinguishing ligand perception and potentially are governed by biased agonism. This emerging concept in GPCR-mediated signal transduction [9] emphasizes that ligands selectively stabilize specific receptor conformations that ultimately favor one (or more) signaling pathways out of the many the receptor is linked to. The final cellular response elicited by the receptor/ligand interaction is therefore biased towards specific signaling pathways [9,10,11]. We also considered that the different classes of agonists might group due to their efficacies and potencies for the same cellular pathways, i.e., apart from the selective activation of entirely different signaling pathways. In this scenario, different classes of agonists would not be associated with their own unique profile of qualitatively distinct cellular responses but instead would cause the same effect, as revealed by similar bias factors, yet on a different scale.

In general, agonist-activated GPCRs stimulate a broad set of heterotrimeric αβγ guanine nucleotide-binding proteins (G proteins), which in turn regulate adenylyl cyclase or phospholipase C (PLC) activities, ultimately leading to effective changes in second messengers cyclic AMP (cAMP) and inositol triphosphate (IP3) levels [12]. Apart from regulating enzymes to control the generation of second messengers, key intracellular signaling pathways are also activated via the GPCR signaling axis. For instance, GPCR-linked stimulation of the mitogen-activated protein kinase (MAPK) pathway, which plays important functional roles in, e.g., the regulation of (chronic) inflammation or even the development of cancer [13,14], is well established. Receptor internalization, which is linked with desensitization and signal termination, is regulated via GPCR/agonist interactions. Notably, emerging novel findings suggest that this mechanism might also be linked to prolonged receptor signaling [15,16].

Here, we compared the signaling profiles of representative microbial, endogenous, and synthetic FPR1 agonists. Our results reveal that the FPR1 activators cluster depending on their origin: the bacterial formylated peptides are strong, potent, and efficacious superagonists, whereas the mitochondrial peptides are less effective. Bias calculation uncovered that although the agonists operate on different levels as defined by their logistic parameters, FPR1 signal transmission generally is biased toward inhibition of cAMP formation.

## 2. Materials and Methods

### 2.1. FPR1 Ligands and Reagents

Formylated peptides corresponding to the N-termini of the human mitochondrially encoded proteins NADH:ubiquinone oxidoreductase core subunit 2 (MT-ND2; fMNPLAQ), NADH:ubiquinone oxidoreductase core subunit 6 (MT-ND6; fMMYALF), and cytochrome b (CYTB; fMTPMRKTNPLMKLIN), the formylated pentapeptide fMIVIL from *Listeria monocytogenes*, and the gG-2p20 peptide GLLWVEVGGEGPGPT derived from the secreted glycoprotein sgG-2 of herpes simplex virus type 2 (HSV-2) were custom-synthesized (Biomatik, Cambridge, ON, Canada). The acetylated peptide Ac2-26 corresponding to the N-terminus of human annexin A1 (AcAMVSEFLKQAWFIENEEQEYVQTVK) and the synthetic agonist W-peptide (WKYMVm) were purchased from Tocris (Wiesbaden-Nordenstadt, Germany). The prototype FPR1 agonist fMLF derived from *E. coli* was purchased from Sigma. Stock solutions were prepared as indicated in Table A1. The mouse monoclonal anti-FLAG antibody M1 (Sigma-Aldrich, Darmstadt, Germany), which recognizes the FLAG epitope only when present at the very N-terminus of the FPR1 receptor, i.e., after successful cleavage of the hemagglutinin signal sequence (see below) in the endoplasmic reticulum (ER), was labeled with DyLight488 antibody labeling kit (Thermo Fisher Scientific, Waltham, MA, USA) according to the manufacturer’s protocol. Pertussis toxin (PTX) from *Bordetella pertussis* was purchased from Tocris, the Gα_q_ inhibitor FR900359 (FR, formerly known as UBO-QIC), a cyclic depsipeptide from the plant *Ardisia crenata sims sims* was purified following a previously published protocol [17]. Reversed-phase high-performance liquid chromatography separation of the FR-containing fraction (column: YMC C_18_ Hydrosphere, 250 × 4.6 mm, 3 μm; MeOH:H_2_O (8:2), 0.7 mL min^−1^) afforded FR with a purity of 95%. For G protein inhibition experiments, cells were pretreated for 16 h with 100 ng/mL PTX or for 1 h with 1 µM FR preincubation in cell culture medium at 37 °C.

### 2.2. FPR1-Encoding Plasmid, HeLa-FPR1- Cell Line, and Cell Culture Conditions

The FPR1 expression vector, containing the N-terminally FLAG-tagged human FPR1, was generated as previously described [18]. The FPR1 coding sequence was PCR-amplified from a cDNA library representing human total leukocyte RNA (Takara Bio, Saint-Germain-en-Laye, France). The FLAG-epitope was introduced immediately upstream to the original FPR1 start codon and is preceded by a cleavable influenza hemagglutinin signal sequence to facilitate cell surface presentation. This tagged FPR1 CDS was transferred into the mammalian expression vector pcDNA3.1 (-) (Thermo Fisher Scientific) via XhoI and EcoRI restriction sites. HeLa cells cultured in Dulbecco’s modified Eagle’s medium (DMEM, Sigma), supplemented with 10% standardized fetal bovine serum (FBS Superior, Biochrom, Cambridge, UK), 100 U/mL penicillin, and 0.1 mg/mL streptomycin) at 37 °C in a 7% CO_2_ atmosphere were transfected using Lipofectamine 2000 (Thermo Fisher Scientific). Clonal lines were selected with 800 ng/mL geneticin (G418, AppliChem, Darmstadt, Germany).

### 2.3. FPR1 Expression Analysis in Parental and Recombinant HeLa Cells by qPCR and Immunofluorescence Microscopy

qRT-PCR was employed to confirm that parental, i.e., non-transfected HeLa cells do not express members of the FPR family at detectable levels and to confirm FPR1 expression in the stably expressing HeLa-FPR1 cell lines. Total RNA from HeLa cells was isolated with the RNeasy mini kit (Qiagen, Hilden, Germany) according to the manufacturers’ instructions; 1 µg of RNA starting material was converted into cDNA with the high-capacity cDNA reverse transcription kit and random hexamer primers (Thermo Fisher Scientific). Subsequent qPCR analysis was performed with QuantiTect primer assays (Qiagen) for FPR1 (Hs_FPR_1_SG, QT00199745) and custom-designed sets of primers (Microsynth, Lindau, Germany) for amplification of FPR2 (for: 5′-TTGGTTTCCCTTTCAACTGG-3′ rev: 5′-AGACGTAAAGCATGGGGTTG-3′) and FPR3 (for: 5′-GGTTGAACGTGTTCATTACC -3′ rev: 5′-TGGTTTCTGTGAATTTTGGC-3′). Housekeeping genes actin (Hs_ACTB_1_SG, QT00095431) and glyceraldehyde 3-phosphate dehydrogenase (Hs_GAPDH_2_SG, QT01192646) served as references. All qPCR reactions were conducted with the Brilliant III Ultra-Fast SYBR Green qPCR Master Mix (Agilent Technologies, Santa Clara, CA, USA). Four independent cell samples were analyzed in technical replicates and amplified for 45 cycles on a CFX 384 real-time PCR cycler. The PCR amplification was analyzed with the CFX Manager Software v.2.1 (Bio-Rad, Hercules, CA, USA).

Expression and correct localization of tagged FPR1 were confirmed by immunofluorescence imaging. HeLa-FPR1 cells were cultured on glass coverslips and fixed with 4% paraformaldehyde for 10 min at room temperature. After incubation with anti-FLAG M1 antibody (diluted 1:100 in 2% BSA in PBS containing Ca^2+^ and Mg^2+^ (PBS^++^, Sigma) for 60 min, cells were treated with anti-mouse Alexa 594-coupled secondary antibody (Invitrogen, Carlsbad, CA, USA) for an additional 45 min at room temperature. To visualize cell nuclei, Hoechst 33,342 stain (Thermo Fisher Scientific, diluted 1:100) was added during the incubation with the secondary antibody. Oregon Green 488 conjugated wheat germ agglutinin (Invitrogen, WGA, 5 µg/mL in Hank’s balanced salt solution containing Ca^2+^ and Mg^2+^ for 10 min at room temperature) was used to label the plasma membrane. Samples were imaged with a LSM800 (Zeiss, Oberkochen, Germany) confocal microscope using a 63× objective.

### 2.4. Flow Cytometric Analysis of Agonist-Induced Receptor Internalization

HeLa-FPR1 cells were treated with vehicle or agonists diluted in internalization medium (IM; DMEM, 20 mM HEPES, 1 mg/mL BSA, pH 7.2) for 15 min at 37 °C. Cells were washed in PBS (Sigma), and detached in PBS/5 mM EDTA for 3 min at 37 °C. For the detection of the cell surface receptor pool, cells were washed with ice-cold PBS containing 5% BSA and 1 mM CaCl_2_ and subsequently incubated with 5 µg/mL DyLight488-conjugated anti-FLAG M1 antibody for 45 min. 7AAD (eBioscience, San Diego, CA, USA) allowed the exclusion of compromised cells. Median fluorescence intensities (MFI) of 10,000 viable cells per condition were measured with a Guava easyCyte flow cytometer and the InCyte^TM^ Software (Merck-Millipore, Darmstadt, Germany). Agonist-induced internalization was defined as the difference between the MFI of vehicle-treated controls and the MFI detected in agonist-treated cells. For each measurement, agonist-induced internalization was normalized to the mean internalization induced by 10^−4^ M W-peptide, which consistently represented the maximum system response for internalization.

### 2.5. HTRF-Based Quantification of cAMP Levels

A competitive immunoassay based on time-resolved measurement of fluorescence resonance energy transfer (HTRF, homogeneous time-resolved fluorescence) between a cryptate-labeled specific antibody (donor) and a d2-coupled cAMP acceptor molecule (cAMP-G_i_ Kit, Cisbio, Codolet, France) was used to measure cyclic AMP (cAMP) formation in cells, as described elsewhere earlier [19]. In brief, cells cultured as described above on 96 well plates (50k cells/well) were incubated with 5 µM of the adenylyl cyclase activator forskolin (Sigma) in complete medium supplemented with 500 µM of the phosphodiesterase inhibitor 3-isobutyl-1-methylxanthine (IBMX, Sigma), together with the agonists at the indicated concentrations, for 30 min at 37 °C. Samples were transferred onto a 384 well low volume plate, conjugates were added, and samples were analyzed with the CLARIOstar reader (BMG Labtech, Ortenberg, Germany) (200 flashes/well, integration start 60 µsec, integration time 400 µsec, settling time 100 µsec). Luminescence signals were expressed as the ratio of 10,000× (acceptor signal/donor signal). Values were normalized to the maximum system output, which was obtained as forskolin-induced cAMP formation.

### 2.6. HTRF-Based Quantification of MAPK/ERK Phosphorylation Levels

The advanced Phospho-ERK1/2 (Thr202/Tyr204) plate-based assay (Cisbio) was used to measure ERK activation through HTRF using a sandwich assay format of a donor-coupled antibody and an acceptor antibody as described before in [20]. Briefly, cells grown on 96 well plates (45k cells/well) were serum-starved for 2 h and stimulated with the agonists at the indicated concentrations for 5 min at 37 °C. Lysates were transferred to a 384 well low volume plate, incubated with the antibodies for 4 h at room temperature, and luminescence was recorded with a CLARIOstar reader (BMG Labtech) (200 flashes/well, integration start 60 µsec, integration time 400 µsec, settling time 100 µsec). HTRF ratios were normalized to maximum system output obtained through stimulation with 100 nM phorbol 12-myristate 13-acetate (PMA).

### 2.7. HTRF-Based Quantification of IP1 Levels

The IP-One-Gq assay (Cisbio), that detects inositol monophosphate, a stable downstream metabolite of IP3 induced by phospholipase C activation, was utilized to establish FR pretreatment conditions, as described elsewhere earlier [21]. In brief, cells grown overnight on 384 well plates (5k cells/well) were pretreated with 1 µM FR for 1 h at 37 °C prior to stimulation with 100 µM phospholipase C activator 2,4,6-trimethyl-N-[3-(trifluoromethyl)phenyl]benzenesulfonamide (m-3M3FBS, Tocris) for 2 h at 37 °C. LiCl present in the stimulation buffer provided in the kit prevented the degradation of IP1. After addition of the conjugates, samples were incubated for 1 h at room temperature and read on a CLARIOstar plate reader (BMG Labtech) (200 flashes/well, integration start 60 µsec, integration time 400 µsec, settling time 100 µsec).

### 2.8. Curve Fitting

For each individual curve, ligand concentrations were log-transformed, normalized, and expressed as fractions of the maximum system response per pathway. Concentration-response curves were analyzed with Graphpad Prism 6 and the in-built four parameters sigmoidal model with a Hill coefficient of 1. EC_50_ values were derived from individual sigmoidal curve fits. If the data fitting was ambiguous without further constraints (very weak partial agonists, no inflection point), the minimum was set to zero and the highest experimental response was considered to represent E_max_. The ROUT method [22] was used to detect and eliminate outliers. An agonist-induced response was defined by a curve span >3 SEM. If no fitting was possible (i.e., no detection of agonist-elicited responses), the ‘no response’ (NR) label was assigned. To analyze the relationship between data, linear regression and Spearman’s correlation coefficients were calculated (Graphpad Prism 8).

Bias was calculated with the operational model [23]. The logarithm of the activity ratio *E*_max_/EC_50_ [24,25,26] served as a surrogate for the actual transduction coefficient log(t/KA) [27] and was calculated individually for each agonist and pathway. To visualize the ligand “texture”, agonist-specific ΔlogR values were plotted for the analyzed pathways. To compare the relative effectiveness of an agonist at a given pathway with the reference agonist, ΔlogR values were calculated as differences of the respective pathway-specific logR of the agonist of interest and the endogenous MT-ND6 peptide as the reference agonist. To rank the relative signaling preferences of a ligand for one pathway over another, ΔΔlogR values were calculated.

## 3. Results

**Figure 1:**
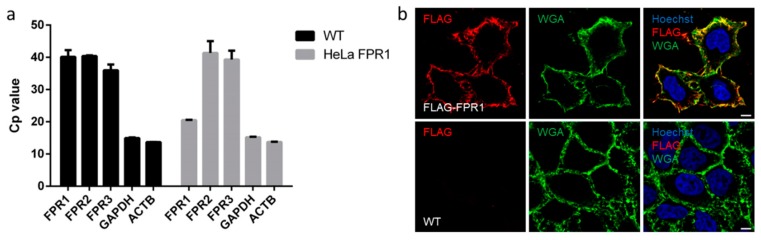
Evaluation of FPR1 (formyl peptide receptor 1) expression and localization in the heterologous HeLa expression system. (**a**) qRT-PCR revealed that none of the three FPR paralogs is expressed in parental HeLa cells (WT), whereas FPR1 is readily detectable in the HeLa-FPR1 transgenic cell line. Housekeeping genes ACTB (beta actin) and GAPDH (glyceraldehyde 3-phosphate dehydrogenase) served as internal controls. (**b**) Confocal immunofluorescence microscopy with the M1 antibody (red channel) confirmed the plasma membrane localization of FLAG-tagged FPR1. The plasma membrane was stained with WGA (green channel), and nuclei were visualized using Hoechst stain (blue channel). Upper panel: HeLa-FPR1 cell line; lower panel: WT HeLa cells, scale bar, 5 μm.

**Figure A1:**
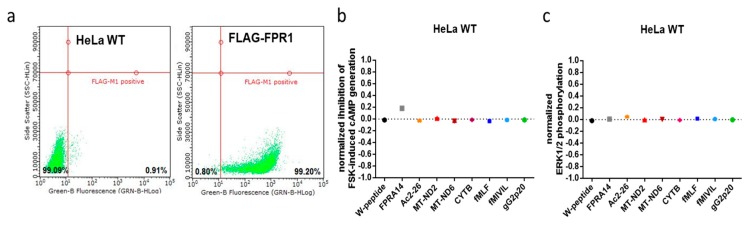
Agonist effects are specific for the FPR1 signaling axis. Parental HeLa cells were subjected to the assays analyzing (**a**) internalization, (**b**) cAMP formation, and (**c**) MAPKinase activation, at agonist concentrations eliciting the respective E_max_ response. (**a**) A representative result, (**b**), (**c**) mean response ± SEM, n 6.

To address in a systematic fashion whether FPR1 agonists differ in their corresponding signaling fingerprints, we established a heterologous FPR1 expression system [18]. The resulting transgenic HeLa-FPR1 cell line was analyzed via qRT-PCR to confirm the stable expression of FPR1 (Figure 1a). As parental HeLa cells did not express any detectable levels of FPR1-3, this experimental design enabled monitoring specific FPR1-mediated responses [8,28]. The correct localization and incorporation of the FLAG-tagged receptor in the plasma membrane were validated by immunofluorescence imaging (Figure 1b). In order to avoid potentially distorting influences resulting from agonist effects other than those elicited via the FPR1 signaling axis, we used parental HeLa cells as a negative control. No signal could be observed when these cells were stained with the M1 antibody, and none of the tested agonists caused any significant response for the analyzed pathways, thereby confirming the specificity of our results (Figure A1).

### 3.1. FRET-Based Analysis of Agonist-Induced Changes in cAMP Levels

**Figure 2:**
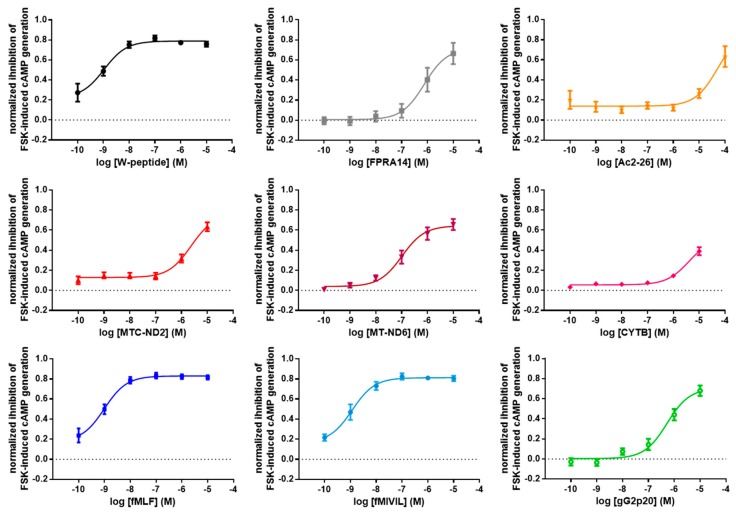
Concentration–response curves for FPR1-mediated inhibition of cAMP production. Cells were stimulated with forskolin (FSK) and the respective FPR1 agonists. Responses recorded 30 min after agonist addition were normalized to the maximum system response obtained with forskolin. Data points represent the mean ± SEM of at least 7 independent measurements.

**Figure A2:**
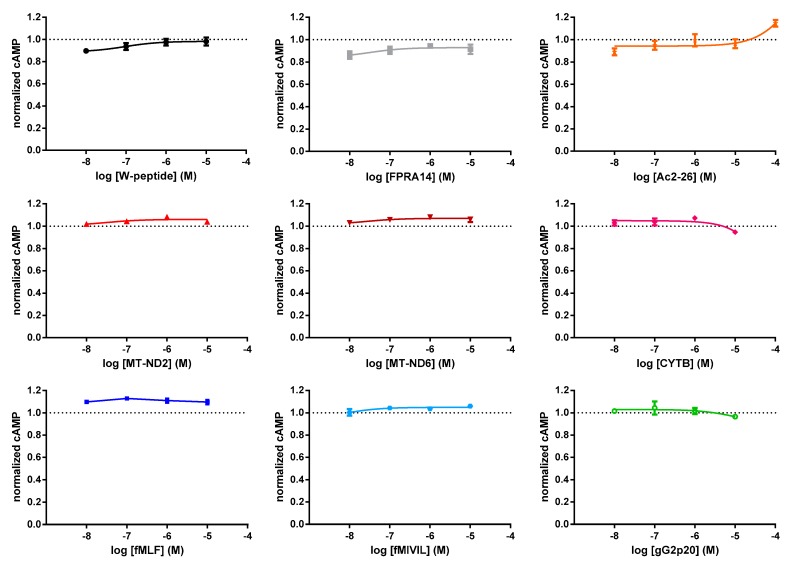
Concentration-response curves for FPR1-mediated stimulation of cAMP production. None of the agonists increased intracellular cAMP levels, in line with the reported FPR1-mediated activation of Gα_i_. Mean response ± SEM, n 3.

**Figure A3:**
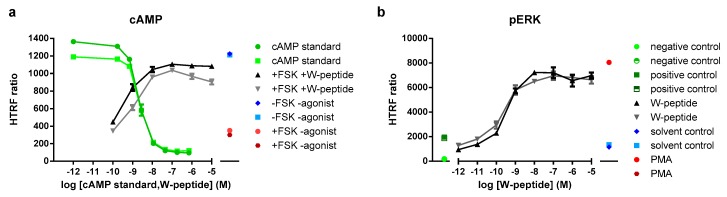
Exemplary raw data of concentration-response curves for FPR1-mediated stimulation of (**a**) cAMP production and (**b**) MAPKinase activation. In addition to the W-peptide measurements, data points for the cAMP standards and the ERK positive and negative controls included in the kit are shown. Data were normalized to the maximum output as obtained with forskolin and PMA.

Agonist-induced cellular responses mediated via FPR1 previously have been reported to depend on Gα_i_ for signal transduction [29]. We, therefore, resorted to a FRET-based system to analyze agonist-mediated changes in cAMP de novo generation. Notably, none of the analyzed FPR1 agonists caused any detectable increase of cellular cAMP levels, thereby confirming that the tested ligands did not trigger FPR1 responses via Gα_s_ (Figure A2). To stimulate the cellular cAMP formation to its maximum, we utilized forskolin, a known activator of adenylyl cyclase activity [30] and determined the inhibitory potential of the different FPR1 agonists (exemplary raw data are shown in Figure A3a). Relative potencies and efficacies of the analyzed agonists differed significantly in our assay (Figure 2). Bacterial peptides [31,32] and the W-peptide [15,33,34], formed a distinct group of agonists with high potencies and efficacies, mitochondrial peptides—on the other hand—were characterized by significantly lower potencies. However, differences in the respective efficacies for bacterial and mitochondrial peptides were not as pronounced. Notably, the annexin A1 peptide Ac2-26 [35], which displayed the lowest potency in our assay, was still able to suppress de novo cAMP formation to a level similar to the mitochondrial agonists. Interestingly, gG2p20, an exogenous ligand-derived from herpes virus [36], and the synthetic small molecule FPRA14 [37,38] mimicked the mitochondrial peptides to some extent. To analyze agonist profiles in more mechanistic detail, we investigated whether the magnitude of the agonist-elicited responses was linked with the respective potencies. Generally, EC_50_ values of the agonists displayed a very strong negative monotonic relationship with their respective ability to elicit E_max_, as demonstrated by the Spearman’s rank correlation coefficient (r = −0.917, *p* = 0.001, n 9).

### 3.2. FRET-Based Analysis of Agonist-Induced Changes in MAPKinase Activation

**Figure 3:**
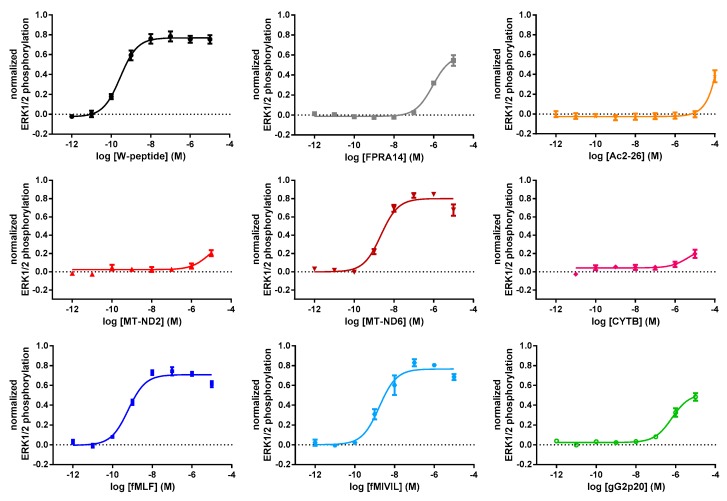
Concentration–response curves for FPR1-mediated phosphorylation of ERK1/2 on Thr202/Tyr204. Responses monitored 5 min after the addition of agonist were normalized to the maximum system response, which was obtained with PMA (phorbol 12-myristate 13-acetate). Data points represent the mean ± SEM of at least 5 independent measurements.

The MAPKinase cascade is a common effector of GPCR activation [39,40,41]. As shown in Figure 3, all agonists were able to increase ERK1/2 phosphorylation (exemplary raw data are shown in Figure A3b). Interestingly, the mitochondrial agonist MT-ND6 performed comparably to the bacterial agonists and the W-peptide, whereas the other mitochondrial peptides, as well as Ac2-26, only weakly activated ERK1/2 phosphorylation. As in the case of the adenylyl cyclase inhibition, gG2p20 and the synthetic small molecule FPRA14 displayed almost identical profiles. Spearman’s rank correlation uncovered a strong negative monotonic correlation between EC_50_ and E_max_ values (r = −0.8, *p* = 0.014, n 9).

### 3.3. Agonist-Mediated Internalization of FPR1

**Figure 4:**
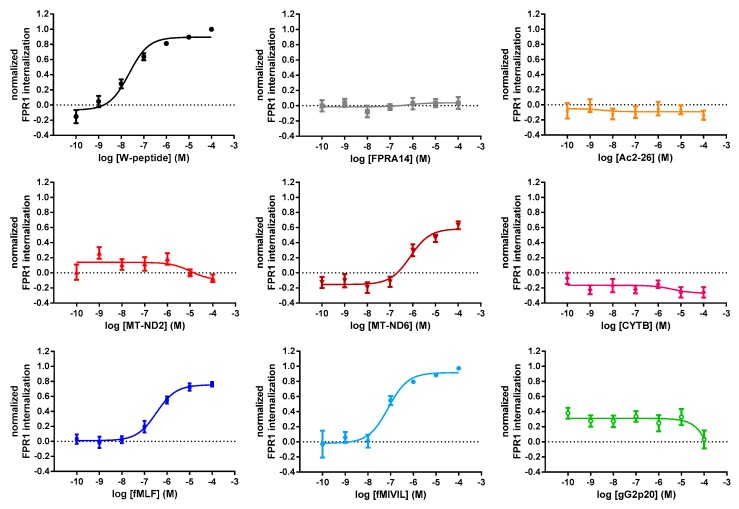
Concentration–response curves for agonist-induced FPR1 internalization. Receptor internalization was calculated as the difference between FLAG signals of unstimulated cells and signals recorded 15 min after agonist addition. Results were normalized to the maximum system response obtained with 10^−4^ M W-peptide. Data points represent the mean ± SEM of 6 independent measurements.

Activated GPCRs usually are removed from the cell surface and are internalized into endosomes [15,16]. To test the potential of our agonists to induce FPR1 internalization, we determined the agonist-dependent decrease in FPR1 cell surface presentation, based on the detection of the N-terminal FLAG-epitope by flow cytometry [42,43]. The actual amount of internalized receptor was calculated via the difference of cell surface signals measured in untreated and agonist-treated samples after 15 min of agonist addition. Administration of W-peptide, bacterial peptides fMLF and fMIVIL, or the endogenous mitochondrial peptide MT-ND6 decreased the cell surface-associated FPR1 pool in a concentration-dependent manner. In contrast to that, the mitochondria-derived peptides MT-ND2, CYTB, the synthetic agonist FPRA14, and the annexin A1 peptide Ac2-26 did not elicit detectable internalization and were therefore deemed non-responders. Importantly, no significant difference was observed between the two formylated ligands fMLF and MT-ND6, indicating that the source of signal (PAMP vs. DAMP) was not encoded by these ligands (Figure 4).

### 3.4. G protein Dependency of Agonist-Mediated Responses

**Figure 5:**
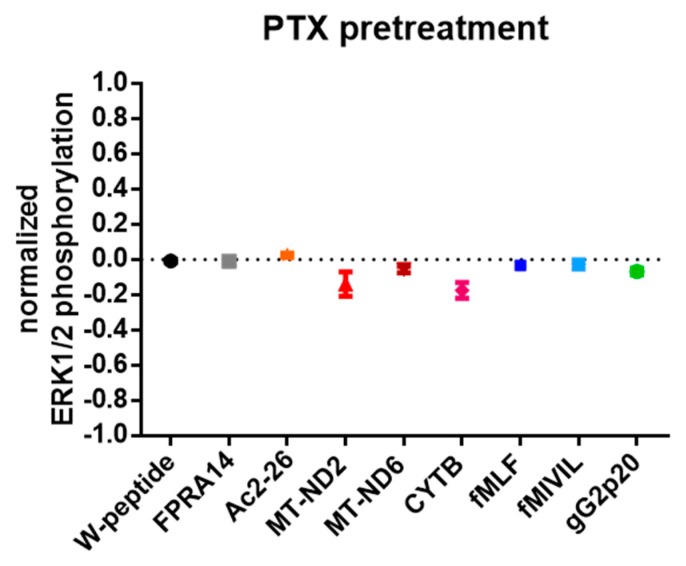
FPR1-mediated ERK1/2 phosphorylation is abolished in PTX (pertussis toxin)-pretreated cells. Cells were pretreated for 16 h with 100 ng/mL PTX and subsequently stimulated with agonist concentrations eliciting the respective E_max_. Results were normalized to the maximum system response obtained with PMA. Data points represent the mean ± SEM of 6 independent measurements.

**Figure 6:**
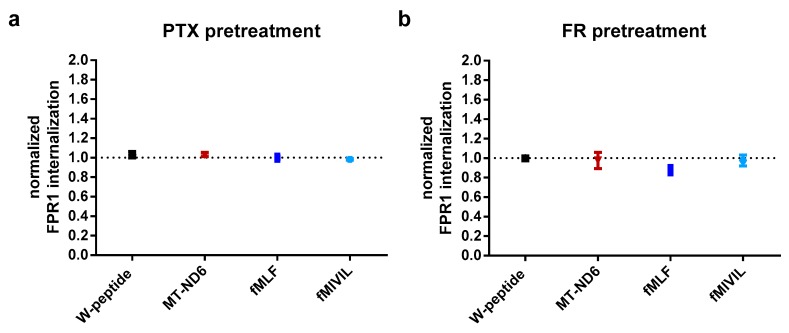
Agonist-induced FPR1 internalization is not dependent on Gα-mediated signal transmission. (**a**) Cells were pretreated either for 16 h with PTX or (**b**) for 1 h with FR and subsequently stimulated with agonist concentrations eliciting the respective E_max_. Results were normalized to the 10^−4^ M W-peptide-induced maximum response. Data points represent the mean ± SEM of 6 independent measurements.

**Figure A4:**
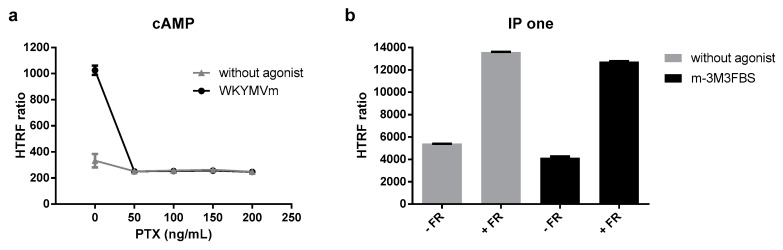
Analysis of G protein dependency. (**a**) PTX-mediated inhibition of FPR1-mediated changes in cAMP generation. (**a**) Maximum FPR1-mediated inhibition of forskolin-induced cAMP formation was completely prevented in cells pretreated for 16 h with 100 ng/mL PTX. (**b**) Effect of 1 µM FR preincubation for 1 h on IP-1 production induced by the phospholipase C activator m-3M3FBS. Data points represent the mean ± SEM.

We considered that the correlation for E_max_ and EC_50_ values for individual agonists and pathways, as revealed by the Spearman’s rank coefficients, might—albeit indirectly—provide insights into common effector proteins interacting with the receptor to transduce agonist-elicited responses. We detected a very strong positive monotonic correlation between EC_50_ values for cAMP inhibition and ERK phosphorylation (r = 0.933, *p* = 0.001, n 9). A similar trend could also be established for the corresponding E_max_ values, which again revealed a strong positive correlation (0.733, *p* = 0.031, n 9; for an overview of logEC_50_ and E_maxA_ values, see Figure A4 and Table A2.). To directly assess whether the different molecular pathways are driven by Gα_i_-protein-dependent signal transduction, we repeated our experiments in the presence of the Gα_i_-specific inhibitor pertussis toxin (PTX) at a concentration that completely abolished the inhibition of cAMP formation induced by W-peptide (Figure A4a). PTX treatment of HeLa-FPR1 cells did not interfere with ERK activation per se, however, agonist-mediated ERK1/2 activation was suppressed, thus strongly implying that both pathways, (at least) in our cellular system, relied on the Gα_i_-protein (Figure 5). In stark contrast, receptor internalization was not dependent on Gα_i_ activation, as agonist-evoked FPR1 internalization was not affected in PTX-pretreated cells (Figure 6a) and was equally undisturbed in cells pretreated with the Gα_q_ inhibitor FR (Figure 6b).

### 3.5. Analysis of Agonist Bias on FPR1

**Figure 7:**
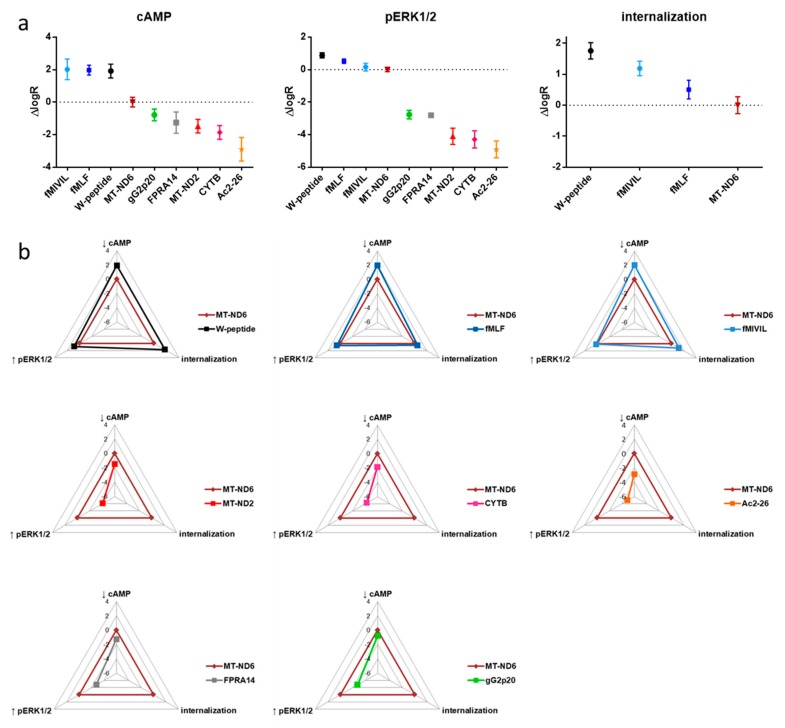
Relative agonist profiles compared to the endogenous reference agonist. (**a**) ΔlogR values were calculated from logR values with the mitochondrial peptide MT-ND6 as reference agonist. Data points represent the mean and 95% confidence intervals. (**b**) Relative agonist activities corresponding to the ΔlogR values for the respective pathways are depicted in radial graphs. Each radius is displayed in the logarithmic scale.

**Figure 8:**
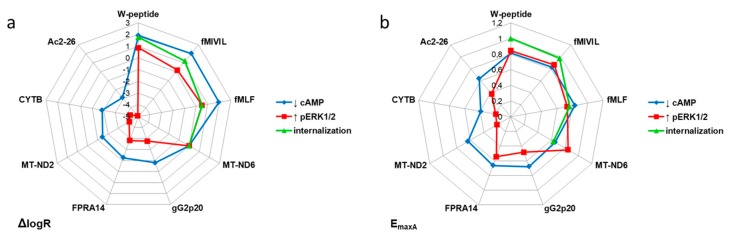
Graphical representation of the agonist profiles. Relative agonist activities corresponding to the ΔlogR values and the logistic parameters (**a**) EC_50_ and (**b**) E_maxA_ for the respective pathways are depicted in radial graphs. Each radius for ΔlogR is displayed in the logarithmic scale, radiuses for E_max_ are scaled linearly.

**Figure 9:**
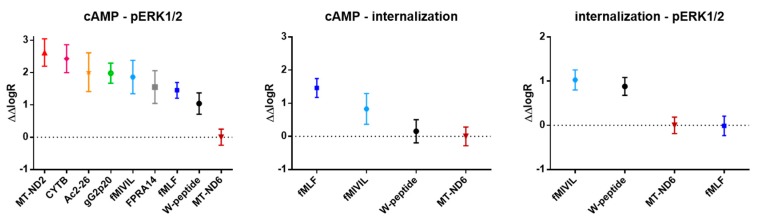
Comparison of agonist pathway preferences. ΔΔlogR values were calculated from ΔlogR values. Data points represent the mean and 95% confidence intervals.

**Figure A5:**
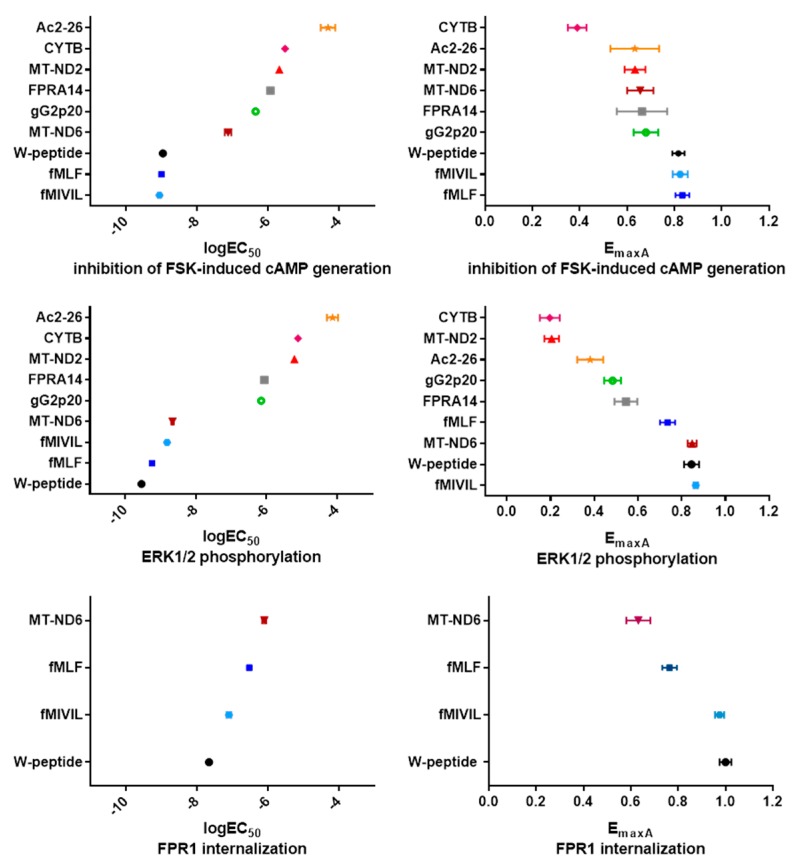
Depiction of logEC_50_ and E_maxA_ values of each pathway. Ligands are arranged according to the intensity of the response they elicited in the respective pathway. Data are expressed as mean ± SEM after outlier removal.

For the identification and analysis of ligand bias [23] associated with the FPR1 signaling axis, we selected the mitochondria-derived MT-ND6 as endogenous reference agonist for all tested pathways. For each agonist and pathway, we determined the corresponding logR (the logarithm of the activity ratio) values [24] and normalized the activities to MT-ND6 (ΔlogR=logR_agonist_-logR_MT-ND6_, see Table I) [23]. Figure 7 summarizes the resulting agonist ranking for each pathway. Overall, two agonist clusters were established: bacterial ligands, along with the W-peptide, which resembles a conserved spatial structure of bacterial agonists [44], constituted the group of “high-performers”. Endogenous mitochondria-derived agonists, the herpes virus-derived peptide, the small synthetic agonist FPRA14, and annexin A1 peptide Ac2-26 represented the second group of considerably lower activity (Figure 7a). Interestingly, the endogenous agonist MT-ND6 featured balanced characteristics, with a profile “in-between” both groups. Graphical representations of the intrinsic activity profiles, i.e., in terms of ΔlogR, further highlight the tendency of our tested ligands to segregate into two clusters, which was observed independently of pathway but in relation to the agonist origin (Figure 7b). However, the segregation was less pronounced when the magnitude of the respective response was taken into consideration (Figure 8, Figure A5, and Table A2). Analysis of pathway preferences, i.e., in terms of ΔlogR values, revealed that FPR1 activation was generally biased towards the inhibition of cAMP formation compared to the activation of the MAPKinase pathway or receptor internalization (Figure 9). However, the direct comparison of the weak agonist Ac2-26 with the strong agonist fMIVIL impressively revealed that bias was realized at different logistic levels (Figure 9).

## 4. Discussion

FPR1 and the corresponding large repertoire of FPR1 agonists, derived from various cellular and pathogenic sources, constitute a powerful system for the detection of insults that are linked with tissue destruction under infectious and sterile conditions, such as pathogenic challenges or trauma caused by burns or injury [9,45]. Because overactivation of the innate immune response is often correlated with excessive and deleterious tissue damage, e.g., in influenza A virus (IAV) infection, targeting the FPR family might represent a novel approach to balance innate immunity. Indeed, our previous studies revealed the advantageous use of the host-derived anti-inflammatory N-terminal annexin A1 peptide Ac-2-26, a pan-FPR agonist [46] to counteract viral load and mortality in a preclinical murine IAV infection model [47], thus encouraging the development of novel FPR-based therapies.

Generally, the often observed signaling diversity elicited by GPCR agonists that are acting on the same receptor is based on the ligand preference for certain receptor conformational states linked to a subset of all possible signaling responses. This diversity has been termed “functional selectivity” and has led to the concept of “biased agonism” and helps to explain different regulatory outcomes via the activation of the same receptor [9,11]. Therapeutically, bias analysis might help to identify compounds that direct receptor signaling toward desired responses, thus aiding in the development of novel therapeutics with an effective pharmacological profile that avoids activation of unwanted signaling pathways and hence side effects [48,49]. Indeed, a few biased agonists have been developed and are currently in various stages of clinical trials; this is true even for FPR-targeting compounds [50].

To further explore the potential of biased agonism acting on FPRs, we selected human FPR1, the founding member of the FPR family [8], for broader analysis. FPR1 represents the sensor platform for short formylated peptides, a pattern commonly associated with bacterial PAMPs and mitochondria-derived DAMPs [51,52]. However, the preference for such modified peptides is not exclusive, and even non-formylated derivatives (such as Ac2-26) are known to activate FPR1 effectively. The structural diversity of FPR1 agonists led us to hypothesize that potentially different classes of agonists might group, based on their origin, i.e., as PAMPs, otherwise endogenous ligands or DAMPs, thus enabling the receptor to decode the actual source of danger and in turn to channel the cellular responses.

Commonly, agonists are classified according to their ability to invoke the maximum receptor-mediated response of a given pathway. These agonists represent “full” agonists, whereas “partial” agonists only elicit a fraction of the cellular responses caused by a full agonist. This classification is intuitive and seems to be well suited to describe the properties of most ligands; it inherently suffers, however, from the disadvantage that potentially better—yet unidentified—agonists (evoking a higher response) cannot be accounted for. An alternative approach classifies agonist efficacies in relation to an endogenous reference agonist of high efficacy. Therefore, some agonists might be identified, which are even capable to elicit stronger cellular responses than those associated with the reference agonist; consequently, these ligands are referred to as “superagonists”, although this term still has to be defined in broader detail [53]. The molecular explanation for this phenomenon might lie in the observation that simultaneous binding of an efficacious agonist and a G protein is required to induce the full receptor response [54,55]. Our results imply that bacterial agonists function as bona fide superagonists at FPR1.

Of note, FPR1 agonist clusters clearly grouped based on ligand efficacies and potencies; these logistic properties were strongly correlated as revealed by the analysis “within” as well as “across” Gα_i_-dependent pathways. Moreover, our results argued in favor of a shared signal transmission selectivity for a given pathway across these structurally unrelated agonist classes. This was most obvious in the case of agonist-mediated ERK activation [56,57], which was entirely G protein-dependent, because in no instance did we observe Gαi-independent activation of the ERK signaling cascade. Yet, lack of evidence for G protein-independent signals, as far as ERK phosphorylation is concerned, does by no means exclude that FPR1 agonists display preferences for supposedly distinct subsets of FPR1-elicited signaling pathways over others. Internalization might—at first glance—appear G protein-independent. However, the most likely scenario probably is that endocytosis requires an active receptor conformation rather than an active signaling pathway and therefore occurs even when G proteins are precluded from active signal transmission. It is therefore perhaps not surprising that particularly high-efficacy ligands are the most effective at causing receptor internalization. It also cannot be ruled out that a given cellular environment is hard-wired for a set of pathways [58]. Hence, the decoding capacity of our heterologous expression system might cause the cells’ inability to distinguish the source of these ligands, leading to so-called “system bias”. Our data suggests that peptide-based FPR1 pharmacotherapy might be worthwhile exploring, however, peptide aggregation is a huge challenge [59].

Based on the bias calculations, all of the agonists preferentially inhibited cAMP over ERK phosphorylation. cAMP is a prominent second messenger in the PKA signaling pathway and a potent regulator of immunity. Because increasing and decreasing cAMP levels are correlated to dampening or stimulating immune responses, respectively, the cellular cAMP balance is considered a bona fide druggable target [60]. Surprisingly, the Ac2-26 peptide was also biased toward adenylyl cyclase inhibition, similar to the endogenous mitochondrial peptides, and the agonistic profile did not resonate with the established anti-inflammatory properties.

The bias in FPR1 activation toward inhibition of cAMP production may signal imminent danger, regardless of the source. However, similar bias factors can be associated with entirely different logistic parameters, and therefore, might cause different levels of physiological response. Our data do not support a selective activation of entirely different signaling pathways, as typically associated with biased agonists. Rather, our findings identified that the actual differences of the danger signals are encoded by the different levels of response (described by the logistic parameters EC_50_ and the maximum agonist-elicited response E_max_).

Another means by which GPCR signaling can be diversified is the organization of GPCR-based signaling platforms via homo- and hetero-oligomerization [61]. Indeed, there is emerging functional evidence for FPR higher-order structures [62,63]. Whether such supramolecular sensor complexes are able to decode additional information is certainly an important line of future research.

## Appendix A

**Table A1:** Ligand stock solutions. Concentrations of stock solutions were calculated based on the solubility of the compound as indicated by the manufacturer and its molecular weight (M). Stock solutions were prepared using the solvents recommended by the manufacturer.

Ligand	Stock Concentration	Solvent	Solubility	M
Ac2-26	324 µM	PBS	1 mg/mL	3089.46 g/mol
CYTB	1.0 mM	DMSO	2 mg/mL	1816.26 g/mol
fMIVIL	3.25 mM	DMSO	2 mg/mL	615.83 g/mol
fMLF	10 mM	DMSO	4 mg/mL	437.60 g/mol
FPRA14	100 mM	DMSO	100 mM	408.92 g/mol
gG2p20	1.36 mM	DMSO	2 mg/mL	1467.62 g/mol
MT-ND2	2.85 mM	DMSO	2 mg/mL	700.79 g/mol
MT-ND6	2.49 mM	DMSO	2 mg/mL	803.00 g/mol
WKYMVm	2.3 mM	H_2_O	2 mg/mL	856.11 g/mol

**Table A2:** Overview of logistic parameters for FPR1 agonists. LogEC_50_ and E_max_ were determined for FPR1 ligands for the analyzed pathways. Data are expressed as mean ± SEM values. NR, no response.

	Ligand	cAMP Formation		ERK1/2 Activation		Internalization	
		logEC_50_	E_maxA_	logEC_50_	E_maxA_	logEC_50_	E_maxA_
synthetic	FPR14	−5.94 0.19	0.66 0.11	−6.06 0.05	0.55 0.05	NR	NR
	W-peptide	−8.96 0.15	0.82 0.03	−9.54 0.06	0.85 0.04	−7.66 0.08	1.00 0.03
endogenous	Ac2-26	−4.31 0.20	0.63 0.10	−4.13 0.16	0.38 0.06	NR	NR
mitochondrial	CYTB	−5.52 0.11	0.39 0.04	−5.11 0.24	0.20 0.05	NR	NR
	MT-ND2	−5.69 0.13	0.63 0.04	−5.21 0.16	0.21 0.03	NR	NR
	MT-ND6	−7.13 0.09	0.66 0.06	−8.66 0.04	0.85 0.02	−6.11 0.06	0.63 0.05
bacterial	fMIVIL	−9.06 0.24	0.82 0.03	−8.81 0.11	0.86 0.01	−7.10 0.06	0.97 0.02
	fMLF	−9.00 0.10	0.83 0.03	−9.24 0.04	0.74 0.04	−6.53 0.10	0.76 0.03
viral	gG2p20	−6.36 0.12	0.68 0.05	−6.15 0.08	0.48 0.04	NR	NR

## References

[1] Takeuchi O., Akira S. (2010). Pattern Recognition Receptors and Inflammation. Cell.

[2] Mogensen T.H. (2009). Pathogen recognition and inflammatory signaling in innate immune defenses. Clin. Microbiol. Rev..

[3] Calfee C.S., Matthay M.A. (2010). Clinical immunology: Culprits with evolutionary ties. Nature.

[4] Dyall S.D., Brown M.T., Johnson P.J. (2004). Ancient invasions: From endosymbionts to organelles. Science (New York N.Y.).

[5] Zhang Q., Raoof M., Chen Y., Sumi Y., Sursal T., Junger W., Brohi K., Itagaki K., Hauser C.J. (2010). Circulating mitochondrial DAMPs cause inflammatory responses to injury. Nature.

[6] Chen G.Y., Nunez G. (2010). Sterile inflammation: Sensing and reacting to damage. Nat. Rev. Immunol..

[7] Le Y., Murphy P.M., Wang J.M. (2002). Formyl-peptide receptors revisited. Trends Immunol..

[8] Boulay F., Tardif M., Brouchon L., Vignais P. (1990). The human N-formylpeptide receptor. Characterization of two cDNA isolates and evidence for a new subfamily of G-protein-coupled receptors. Biochemistry.

[9] Raabe C.A., Groper J., Rescher U. (2019). Biased perspectives on formyl peptide receptors. Biochim. Et Biophys. Acta. Mol. Cell Res..

[10] Rankovic Z., Brust T.F., Bohn L.M. (2016). Biased agonism: An emerging paradigm in GPCR drug discovery. Bioorganic Med. Chem. Lett..

[11] Wootten D., Christopoulos A., Marti-Solano M., Babu M.M., Sexton P.M. (2018). Mechanisms of signalling and biased agonism in G protein-coupled receptors. Nat. Rev. Mol. Cell Biol..

[12] Flock T., Hauser A.S., Lund N., Gloriam D.E., Balaji S., Babu M.M. (2017). Selectivity determinants of GPCR-G-protein binding. Nature.

[13] Jain R., Watson U., Vasudevan L., Saini D.K. (2018). ERK Activation Pathways Downstream of GPCRs. Int. Rev. Cell Mol. Biol..

[14] Huang P., Han J., Hui L. (2010). MAPK signaling in inflammation-associated cancer development. Protein Cell.

[15] Calebiro D., Godbole A. (2018). Internalization of G-protein-coupled receptors: Implication in receptor function, physiology and diseases. Best Pract. Res. Clin. Endocrinol. Metab..

[16] Pavlos N.J., Friedman P.A. (2017). GPCR Signaling and Trafficking: The Long and Short of It. Trends Endocrinol. Metab..

[17] Schrage R., Schmitz A.L., Gaffal E., Annala S., Kehraus S., Wenzel D., Bullesbach K.M., Bald T., Inoue A., Shinjo Y. (2015). The experimental power of FR900359 to study Gq-regulated biological processes. Nat. Commun..

[18] Ernst S., Zobiack N., Boecker K., Gerke V., Rescher U. (2004). Agonist-induced trafficking of the low-affinity formyl peptide receptor FPRL1. Cell. Mol. Life Sci..

[19] Conche C., Boulla G., Trautmann A., Randriamampita C. (2009). T cell adhesion primes antigen receptor-induced calcium responses through a transient rise in adenosine 3′,5′-cyclic monophosphate. Immunity.

[20] Grundmann M., Merten N., Malfacini D., Inoue A., Preis P., Simon K., Ruttiger N., Ziegler N., Benkel T., Schmitt N.K. (2018). Lack of beta-arrestin signaling in the absence of active G proteins. Nat. Commun..

[21] Gao Z.G., Jacobson K.A. (2016). On the selectivity of the Galphaq inhibitor UBO-QIC: A comparison with the Galphai inhibitor pertussis toxin. Biochem. Pharmacol..

[22] Motulsky H.J., Brown R.E. (2006). Detecting outliers when fitting data with nonlinear regression-a new method based on robust nonlinear regression and the false discovery rate. BMC Bioinform..

[23] Kenakin T., Watson C., Muniz-Medina V., Christopoulos A., Novick S. (2012). A simple method for quantifying functional selectivity and agonist bias. ACS Chem. Neurosci..

[24] Ehlert F.J. (2005). Analysis of allosterism in functional assays. J. Pharmacol. Exp. Ther..

[25] Tran J.A., Chang A., Matsui M., Ehlert F.J. (2009). Estimation of relative microscopic affinity constants of agonists for the active state of the receptor in functional studies on M2 and M3 muscarinic receptors. Mol. Pharmacol..

[26] Figueroa K.W., Griffin M.T., Ehlert F.J. (2009). Selectivity of agonists for the active state of M1 to M4 muscarinic receptor subtypes. J. Pharmacol. Exp. Ther..

[27] Griffin M.T., Figueroa K.W., Liller S., Ehlert F.J. (2007). Estimation of agonist activity at G protein-coupled receptors: Analysis of M2 muscarinic receptor signaling through Gi/o,Gs, and G15. J. Pharmacol. Exp. Ther..

[28] Muto Y., Guindon S., Umemura T., Kohidai L., Ueda H. (2015). Adaptive evolution of formyl peptide receptors in mammals. J. Mol. Evol..

[29] Tsu R.C., Lai H.W., Allen R.A., Wong Y.H. (1995). Differential coupling of the formyl peptide receptor to adenylate cyclase and phospholipase C by the pertussis toxin-insensitive Gz protein. Biochem. J..

[30] Seamon K.B., Padgett W., Daly J.W. (1981). Forskolin: Unique diterpene activator of adenylate cyclase in membranes and in intact cells. Proc. Natl. Acad. Sci. USA.

[31] Marasco W.A., Phan S.H., Krutzsch H., Showell H.J., Feltner D.E., Nairn R., Becker E.L., Ward P.A. (1984). Purification and identification of formyl-methionyl-leucyl-phenylalanine as the major peptide neutrophil chemotactic factor produced by Escherichia coli. J. Biol. Chem..

[32] Southgate E.L., He R.L., Gao J.L., Murphy P.M., Nanamori M., Ye R.D. (2008). Identification of formyl peptides from Listeria monocytogenes and Staphylococcus aureus as potent chemoattractants for mouse neutrophils. J. Immunol. (Baltim. Md. 1950).

[33] Bae Y.S., Kim Y., Park J.C., Suh P.G., Ryu S.H. (2002). The synthetic chemoattractant peptide, Trp-Lys-Tyr-Met-Val-D-Met, enhances monocyte survival via PKC-dependent Akt activation. J. Leukoc. Biol..

[34] Katada T., Ui M. (1976). Accelerated turnover of blood glucose in pertussis-sensitized rats due to combined actions of endogenous insulin and adrenergic beta-stimulation. Biochim. Et Biophys. Acta.

[35] Perretti M., Ahluwalia A., Harris J.G., Goulding N.J., Flower R.J. (1993). Lipocortin-1 fragments inhibit neutrophil accumulation and neutrophil-dependent edema in the mouse. A qualitative comparison with an anti-CD11b monoclonal antibody. J. Immunol. (Baltim. Md. 1950).

[36] Bellner L., Thoren F., Nygren E., Liljeqvist J.A., Karlsson A., Eriksson K. (2005). A proinflammatory peptide from herpes simplex virus type 2 glycoprotein G affects neutrophil, monocyte, and NK cell functions. J. Immunol. (Baltim. Md. 1950).

[37] Cussell P.J.G., Howe M.S., Illingworth T.A., Gomez Escalada M., Milton N.G.N., Paterson A.W.J. (2019). The formyl peptide receptor agonist FPRa14 induces differentiation of Neuro2a mouse neuroblastoma cells into multiple distinct morphologies which can be specifically inhibited with FPR antagonists and FPR knockdown using siRNA. PLoS ONE.

[38] Schepetkin I.A., Kirpotina L.N., Khlebnikov A.I., Quinn M.T. (2007). High-throughput screening for small-molecule activators of neutrophils: Identification of novel N-formyl peptide receptor agonists. Mol. Pharmacol..

[39] Lopez-Ilasaca M. (1998). Signaling from G-protein-coupled receptors to mitogen-activated protein (MAP)-kinase cascades. Biochem. Pharmacol..

[40] Goldsmith Z.G., Dhanasekaran D.N. (2007). G protein regulation of MAPK networks. Oncogene.

[41] Gutkind J.S. (2000). Regulation of mitogen-activated protein kinase signaling networks by G protein-coupled receptors. Sci. Stke Signal Transduct. Knowl. Environ..

[42] Vines C.M., Revankar C.M., Maestas D.C., LaRusch L.L., Cimino D.F., Kohout T.A., Lefkowitz R.J., Prossnitz E.R. (2003). N-formyl peptide receptors internalize but do not recycle in the absence of arrestins. J. Biol. Chem..

[43] Wagener B.M., Marjon N.A., Prossnitz E.R. (2016). Regulation of N-Formyl Peptide Receptor Signaling and Trafficking by Arrestin-Src Kinase Interaction. PLoS ONE.

[44] Bufe B., Zufall F. (2016). The sensing of bacteria: Emerging principles for the detection of signal sequences by formyl peptide receptors. Biomol. Concepts.

[45] He H.Q., Ye R.D. (2017). The Formyl Peptide Receptors: Diversity of Ligands and Mechanism for Recognition. Mol. (BaselSwitz.).

[46] Ernst S., Lange C., Wilbers A., Goebeler V., Gerke V., Rescher U. (2004). An annexin 1 N-terminal peptide activates leukocytes by triggering different members of the formyl peptide receptor family. J. Immunol. (Baltim. Md. 1950).

[47] Schloer S., Hubel N., Masemann D., Pajonczyk D., Brunotte L., Ehrhardt C., Brandenburg L.O., Ludwig S., Gerke V., Rescher U. (2019). The annexin A1/FPR2 signaling axis expands alveolar macrophages, limits viral replication, and attenuates pathogenesis in the murine influenza A virus infection model. Off. Publ. Fed. Am. Soc. Exp. Biol..

[48] Kenakin T. (2019). Biased Receptor Signaling in Drug Discovery. Pharmacol. Rev..

[49] Gesty-Palmer D., Luttrell L.M. (2011). Refining efficacy: Exploiting functional selectivity for drug discovery. Adv. Pharmacol. (San DiegoCalif.).

[50] Qin C.X., May L.T., Li R., Cao N., Rosli S., Deo M., Alexander A.E., Horlock D., Bourke J.E., Yang Y.H. (2017). Small-molecule-biased formyl peptide receptor agonist compound 17b protects against myocardial ischaemia-reperfusion injury in mice. Nat. Commun..

[51] Meyer A., Laverny G., Bernardi L., Charles A.L., Alsaleh G., Pottecher J., Sibilia J., Geny B. (2018). Mitochondria: An Organelle of Bacterial Origin Controlling Inflammation. Front. Immunol..

[52] Rabiet M.J., Huet E., Boulay F. (2005). Human mitochondria-derived N-formylated peptides are novel agonists equally active on FPR and FPRL1, while Listeria monocytogenes-derived peptides preferentially activate FPR. Eur. J. Immunol..

[53] Schrage R., De Min A., Hochheiser K., Kostenis E., Mohr K. (2016). Superagonism at G protein-coupled receptors and beyond. Br. J. Pharmacol..

[54] Rasmussen S.G., DeVree B.T., Zou Y., Kruse A.C., Chung K.Y., Kobilka T.S., Thian F.S., Chae P.S., Pardon E., Calinski D. (2011). Crystal structure of the beta2 adrenergic receptor-Gs protein complex. Nature.

[55] Rasmussen S.G., Choi H.J., Fung J.J., Pardon E., Casarosa P., Chae P.S., Devree B.T., Rosenbaum D.M., Thian F.S., Kobilka T.S. (2011). Structure of a nanobody-stabilized active state of the beta(2) adrenoceptor. Nature.

[56] Pyne N.J., Pyne S. (2011). Receptor tyrosine kinase-G-protein-coupled receptor signalling platforms: Out of the shadow?. Trends Pharmacol. Sci..

[57] Eishingdrelo H., Kongsamut S. (2013). Minireview: Targeting GPCR Activated ERK Pathways for Drug Discovery. Curr. Chem. Genom. Transl. Med..

[58] Malhotra D., Shin J., Solnica-Krezel L., Raz E. (2018). Spatio-temporal regulation of concurrent developmental processes by generic signaling downstream of chemokine receptors. eLife.

[59] Zapadka K.L., Becher F.J., Gomes Dos Santos A.L., Jackson S.E. (2017). Factors affecting the physical stability (aggregation) of peptide therapeutics. Interface Focus.

[60] Raker V.K., Becker C., Steinbrink K. (2016). The cAMP Pathway as Therapeutic Target in Autoimmune and Inflammatory Diseases. Front. Immunol..

[61] Smith N.J., Milligan G. (2010). Allostery at G protein-coupled receptor homo- and heteromers: Uncharted pharmacological landscapes. Pharmacol. Rev..

[62] Kasai R.S., Suzuki K.G., Prossnitz E.R., Koyama-Honda I., Nakada C., Fujiwara T.K., Kusumi A. (2011). Full characterization of GPCR monomer-dimer dynamic equilibrium by single molecule imaging. J. Cell Biol..

[63] Cooray S.N., Gobbetti T., Montero-Melendez T., McArthur S., Thompson D., Clark A.J., Flower R.J., Perretti M. (2013). Ligand-specific conformational change of the G-protein-coupled receptor ALX/FPR2 determines proresolving functional responses. Proc. Natl. Acad. Sci. USA.

